# Guidelines for autopsy investigation of sudden cardiac death: 2017 update from the Association for European Cardiovascular Pathology

**DOI:** 10.1007/s00428-017-2221-0

**Published:** 2017-09-09

**Authors:** Cristina Basso, Beatriz Aguilera, Jytte Banner, Stephan Cohle, Giulia d’Amati, Rosa Henriques de Gouveia, Cira di Gioia, Aurelie Fabre, Patrick J. Gallagher, Ornella Leone, Joaquin Lucena, Lubov Mitrofanova, Pilar Molina, Sarah Parsons, Stefania Rizzo, Mary N. Sheppard, Maria Paz Suárez Mier, S. Kim Suvarna, Gaetano Thiene, Allard van der Wal, Aryan Vink, Katarzyna Michaud, on behalf of the Association for European Cardiovascular Pathology

**Affiliations:** 1 Cardiovascular Pathology, Department of Cardiac, Thoracic and Vascular Sciences, University of Padua, Padua, Italy; 2 Histopathology Service, National Institute of Toxicology and Forensic Sciences, Madrid, Spain; 3 Department of Forensic Medicine, University of Copenhagen, Copenhagen, Denmark; 4 Department of Pathology and Laboratory Medicine, Grand Rapids, MI USA; 5 Department of Radiological, Oncological and Pathological Sciences, Sapienza, University of Rome, Rome, Italy; 6 Department of Pathology, Hospital de Santa Cruz (CHLO), Lisbon & Forensic Pathology, INMLCF & FMUC, Coimbra, Portugal; 7 Department of Histopathology, St Vincent’s University Hospital, University College Dublin School of Medicine, Dublin, Ireland; 8 Medical Education, Bristol University, Bristol, UK; 9 Department of Pathology, Sant’Orsola-Malpighi University Hospital, Bologna, Italy; 10 Forensic Pathology Service, Institute of Legal Medicine and Forensic Sciences, Seville, Spain; 11 Department of Pathology, Federal Almazov North-West Medical Research Centre, St. Petersburg, Russian Federation; 12 Forensic Pathology Service, Institute of Legal Medicine and Forensic Sciences, Valencia, Spain; 13 Victorian Institute of Forensic Medicine and Monash University, Victoria, Australia; 14 Department of Cardiovascular Pathology, St Georges Medical School, London, UK; 15 Sheffield Teaching Hospitals, Sheffield, UK; 16 Pathology, Academic Medical Center, University of Amsterdam, Amsterdam, Netherlands; 17 Department of Pathology, University Medical Center Utrecht, Utrecht, Netherlands; 18 University Hospital of Lausanne, University Center of Legal Medicine, Lausanne and Geneva, Chemin de la Vulliette 4, 25, 1000 Lausanne, Switzerland

**Keywords:** Autopsy, Guidelines, Protocol, Sudden cardiac death

## Abstract

Although sudden cardiac death (SCD) is one of the most important modes of death in Western countries, pathologists and public health physicians have not given this problem the attention it deserves. New methods of preventing potentially fatal arrhythmias have been developed and the accurate diagnosis of the causes of SCD is now of particular importance. Pathologists are responsible for determining the precise cause and mechanism of sudden death but there is still considerable variation in the way in which they approach this increasingly complex task. The Association for European Cardiovascular Pathology has developed these guidelines, which represent the minimum standard that is required in the routine autopsy practice for the adequate investigation of SCD. The present version is an update of our original article, published 10 years ago. This is necessary because of our increased understanding of the genetics of cardiovascular diseases, the availability of new diagnostic methods, and the experience we have gained from the routine use of the original guidelines. The updated guidelines include a detailed protocol for the examination of the heart and recommendations for the selection of histological blocks and appropriate material for toxicology, microbiology, biochemistry, and molecular investigation. Our recommendations apply to university medical centers, regionals hospitals, and all healthcare professionals practicing pathology and forensic medicine. We believe that their adoption throughout Europe will improve the standards of autopsy practice, allow meaningful comparisons between different communities and regions, and permit the identification of emerging patterns of diseases causing SCD. Finally, we recommend the development of regional multidisciplinary networks of cardiologists, geneticists, and pathologists. Their role will be to facilitate the identification of index cases with a genetic basis, to screen appropriate family members, and ensure that appropriate preventive strategies are implemented.

## Introduction

The present document is an update of the 2008 Guidelines for autopsy investigation of sudden cardiac death (SCD) developed by the Association for European Cardiovascular Pathology (AECVP) [1]. The update is timely in light of the new developments and increased availability of diagnostic tools at post-mortem and the growing awareness that a significant proportion of SCD, particularly at young age, are due to heredo-familial diseases. The writing committee is composed of clinical and forensic pathologists whose collective interests and experience cover the full range of disorders causing SCD. An extensive literature survey was conducted and all members of the writing committee approved the guidelines.

## Definitions and epidemiology

Sudden death (SD) is often the first clinical manifestation of an underlying disease in previously asymptomatic, apparently “healthy” subjects. In this setting, autopsy represents the first, and only, opportunity to establish and register an accurate cause of death. In recent decades, there has been a substantial worldwide decrease in autopsy rates. As a result, important information may not reach national health registries and essential investigations and preventative care may not be implemented [2–4].

The major difficulties in interpreting epidemiological data are the lack of standardization in death certificate coding and the variability in the definition of SD and SCD. SCD has been defined as “a natural, unexpected fatal event occurring within one hour from the onset of symptoms in an apparently healthy subject or in one whose disease was not so severe as to predict an abrupt outcome” [5]. This is a good description of many witnessed deaths in the community or emergency departments. It is less satisfactory in pathological practice, especially forensic practice, where autopsies may be requested on patients whose deaths were not witnessed, occurred during sleep, or at an unknown time before their bodies were discovered. Under these circumstances, it is probably reasonable to assume that death was sudden if the deceased was known to be in good health 24 h before death occurred [6]. Furthermore, for practical purposes, a death can also be classified as sudden if a patient was resuscitated after a cardiac arrest, survived on life support for a limited period, and then died due to irreversible brain damage.

Other definitions commonly used by cardiologists include sudden arrhythmic death syndrome (SADS) when both autopsy and toxicology investigations are inconclusive, the heart is structurally normal at gross and histological examination, and non-cardiac etiologies are excluded (in infants it is called sudden infant death syndrome, SIDS). Sudden unexplained death syndrome (SUDS) is used when death is without an apparent cause and an autopsy has *not* been performed (in infants it is called sudden unexplained death in infancy, SUDI) [7]. However, clear contradictions arise when comparing the meaning of these terms with that accepted in common forensic practice, where the death is considered as unexplained only by exclusion, i.e., after adequate post-mortem investigation including autopsy, death scene investigation, and the evaluation of the medical history of the victim and his/her family and of the circumstances of death.

Worldwide cardiovascular diseases are considered to be responsible for approximately 17 million deaths every year and about 25% are SCD [8]. A recent systematic review of publications on the incidence of SCD in the USA demonstrated that very few studies used primary data, that is data collected directly from first-hand experience. In addition, definitions of SCD and cardiac arrest are not standardized across the medical community [9]. The incidence of SCD increases dramatically with age, in parallel with the age-related increase of coronary heart disease. In adolescents and young adults (<35 years), the approximate incidence is 0.01 per 1000 per year and cardiomyopathies, myocarditis, premature coronary artery disease, congenital coronary artery anomalies, and channelopathies play a major causative role [10–12]. The incidence of SCD then increases, reaching about 1 per 1000 per year in the subjects 35–40 years, 2 per 1000 per year by 60 years, and 200 per 1000 per year in the elderly [13, 14]. Of course, the distribution of risk varies according to clinical and population profiles. The overall estimate in the population is in the range of 300,000–350,000 SCDs per year in the USA [15, 16]. Event rates in Europe are similar to those in the USA [17], but with significant geographic variations. The risk increases in higher-risk subgroups. For instance, the SCD incidence is 0.1–0.2 per 1000 per year in subjects with a high coronary risk profile, 0.5 per 1000 per year in those with a prior coronary event, 1.5 per 1000 per year in those with congestive heart failure and ejection fraction (EF) < 35%, and 2.5 per 1000 per year in cardiac arrest survivors. A combination of prior myocardial infarction, low EF and ventricular tachycardia, is associated with a SCD risk of nearly 3.5 per 1000 per year [18] . However, nearly two third of SCDs occur as the first clinical manifestation of cardiac disease or, in the setting of known cardiac disease, in the absence of risk predictors.

## Autopsies in SD cases

Pathologists are responsible for establishing the precise cause of SD but there is considerable variation in the way in which they approach this increasingly complex task. A variety of book chapters, professional guidelines, and articles have described how pathologists should investigate SD. However, there is little consistency between centers, even in individual countries. We describe the minimum standard that is required in the routine autopsy practice for the adequate assessment of SCD in the general population, *excluding SIDS* ***.***

Autopsies in SD cases are generally performed in two distinct settings, the clinical autopsy and the forensic autopsy. The clinical autopsy is performed at the request of the physician of the deceased or even the family in some countries depending on the national law. In cases of SD, the goal of the clinical autopsy is to determine whether death was due to cardiac disease or one of the many non-cardiac causes. The clinical autopsy generally includes histology. Despite important advances over the last 10 years, ancillary examinations, such as toxicology, microbiology, and sampling for genetics are, all too often, not adequately performed. The forensic autopsy is performed in the case of unexpected, unexplained, or alleged unnatural deaths. A forensic autopsy may be requested by a magistrate, public prosecutor, official death investigator, coroner, or the police to assist in determining the cause and manner of death. Considerable variations exist regarding the legal aspects of authorization and consent in different countries, even though the norm for the harmonization of medico-legal autopsies was established by the European Union in 1999 [19].

## The role of the autopsy in SD

To establish or consider:

- whether death is attributable to a *cardiac disease* or to other causes of SD;
- the nature of the cardiac disease, and whether the mechanism was arrhythmic or mechanical;
- whether the condition causing SD may be inherited, requiring screening and counseling of the next of kin
- the possibility of toxic or illicit drug abuse, trauma, and other unnatural causes;
- the role of third persons in the death.

## Clinical information relevant to the autopsy

In practice, the amount of information that is available before autopsy is variable. Information from family members or the general practitioner of the deceased can be especially valuable. Ideally, the following information is required:

- Age, gender, occupation, lifestyle (especially alcohol or smoking), usual pattern of exercise or athletic activity;
- Circumstances of death: date, time interval (instantaneous or number of hours after onset of symptoms), place of death (at home, at work, in hospital, at recreation), circumstances (at rest, during sleep, during exercise, and whether athletic or non-athletic, during emotional stress), witnessed or un-witnessed, any suspicious circumstances (immersion, traffic accident, intoxication, etc.);
- Past medical history: general health status, previous significant illnesses (especially syncope, chest pain, and palpitations, particularly during exercise, hypertension, respiratory and recent infectious disease, epilepsy, asthma, etc.), previous surgical operations or interventions, previous ECG tracings and chest X-rays, results of cardiovascular examination, laboratory investigations (especially lipid profiles); congenital deafness, periodic paralysis, calcium and potassium metabolism alterations, or any other relevant medical history, not related to the cardiovascular system, that could raise the suspicion of a systemic disease with cardiac involvement;
- Previous cardiovascular intervention: the pathologists should discuss with the surgeon and interventional cardiologist before, during, or after the autopsy. The surgical and interventional report should be always obtained before the autopsy and/or heart examination;
- Prescription and non-prescription medications;
- Family cardiac history: ischemic heart disease and premature SD, arrhythmias, inherited cardiovascular diseases, unexplained syncope, epilepsy, pacemaker or ICD (implantable cardioverter defibrillator), heart transplant;
- ECG tracing taken during resuscitation, serum enzyme, and troponin measurements.

## The autopsy procedure

All SD autopsies should be sequential structured examinations. They should specifically address the major causes of extra-cardiac and cardiac SD. All aspects of the autopsy procedure should adhere to the Recommendations on the Harmonisation of Medico-Legal Autopsy Rules produced by the Committee of Ministers of the Council of Europe [19] .

### External examination of the body

- Establish body weight and height (to correlate with heart weight and wall thicknesses) [20–23]. Measurement of the waist circumference of the cadaver and calculation of the so-called waist-to-height ratio has been also proposed [24].
- Look for any dysmorphic feature, skin, hair, skeletal abnormalities, etc.
- At a forensic autopsy, all external and internal injuries are studied and described in detail and are usually photographed. In some departments, CT and MR imaging are performed before post-mortem and this practice may become more common.
- Check for recent intravenous access, intubation, ECG pads, defibrillator and electrical burns, drain sites, and traumatic lesions.
- Check for ICD/pacemaker [25]; see Medicines and Healthcare products Regulatory Agency 2008 notice for safe removal and interrogation [26].

### Full autopsy, with sequential approach to the causes of SD

#### Exclusion of non-cardiac causes of natural SD

Any natural SD can be considered cardiac in origin after exclusion of non-cardiac causes. Thus, a full autopsy with sequential approach should be always performed to exclude common and uncommon extra-cardiac causes of SD, especially:

- Cerebral (e.g., subarachnoid or intracerebral hemorrhage)
- Respiratory (e.g., asthma, bronchopneumonia)
- Acute hemorrhagic shock (e.g., ruptured extra-pericardial aortic aneurysm, peptic ulcer)
- Septic shock (Waterhouse-Friderichsen syndrome)

#### Search for cardiac causes of SD

Many cardiovascular diseases can cause SCD, either through an arrhythmic mechanism (electric SCD) or by compromising the mechanical function of the heart and great vessels (mechanical SCD). These disorders may affect the coronary arteries, the myocardium, the cardiac valves, the conducting system, the intra-pericardial aorta, or the pulmonary artery.

##### The standard gross examination of the heart

**Fig. 1:**
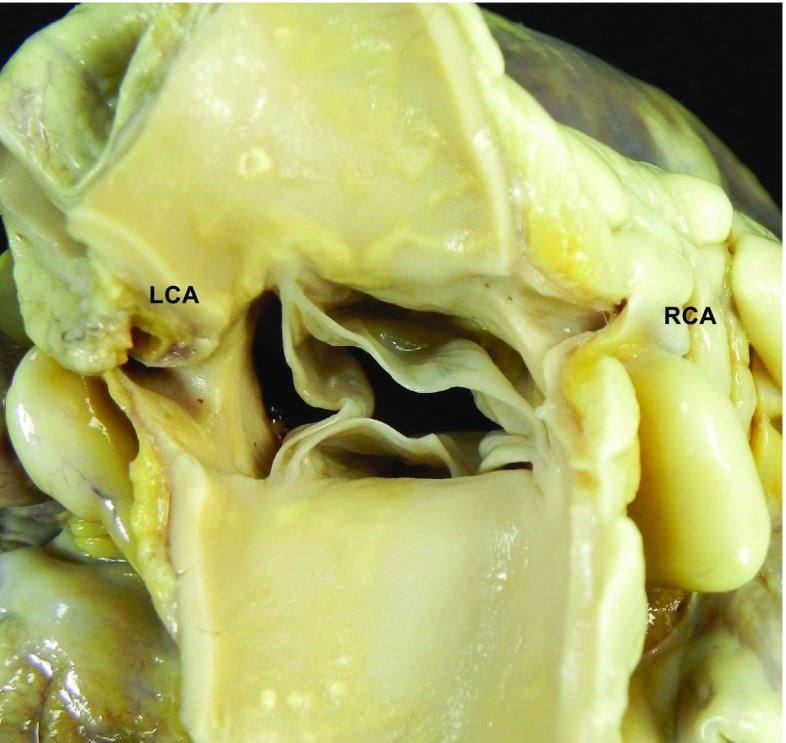
After a transection of the aorta 3 cm above the aortic valve, inspection of the coronary ostia location in the proper sinus (LCA=left coronary artery; RCA= right coronary artery)

**Fig. 2:**
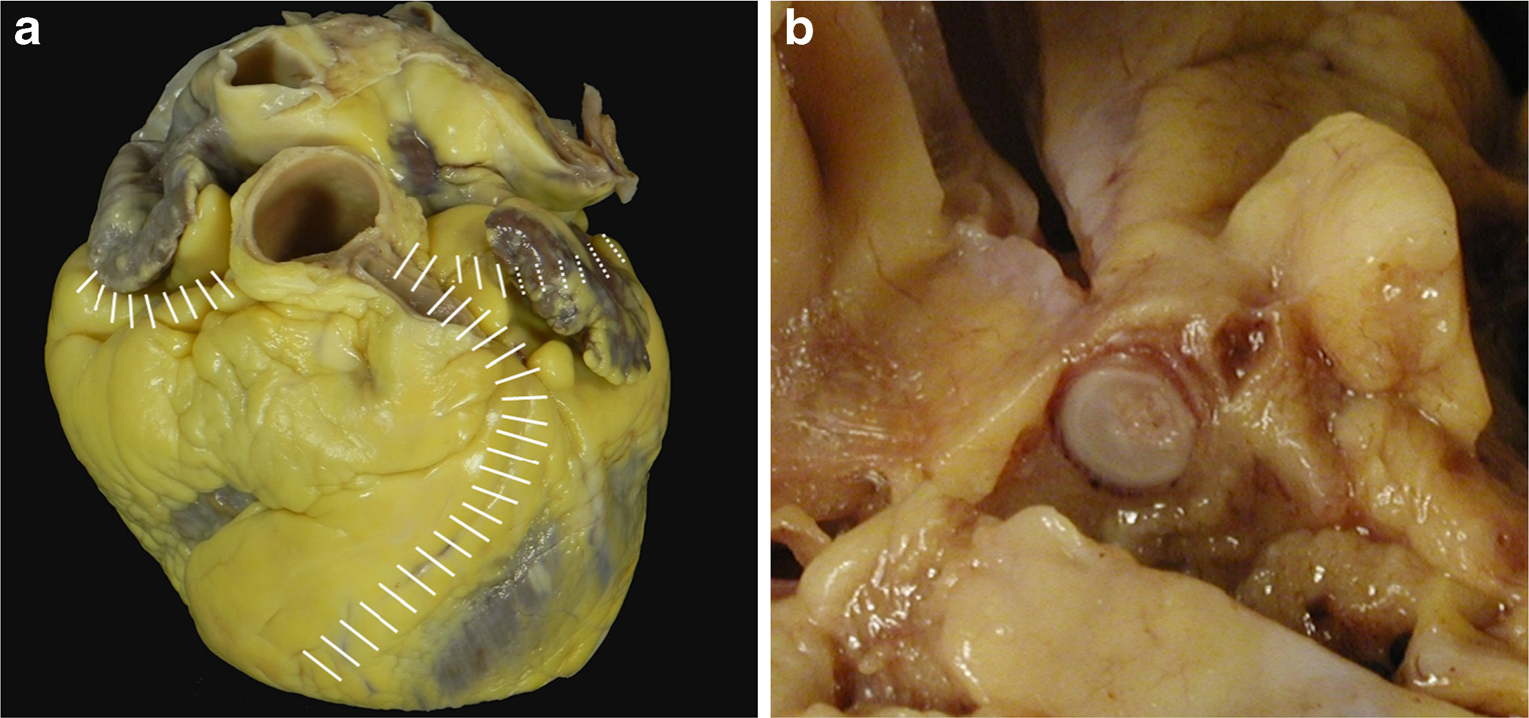
**a** Schematic representation of serial cross sectioning of the sub-epicardial coronary artery tree. **b** A careful inspection of the serial sections is needed not to miss any coronary lesion. A transverse cut of the first tract of the left anterior descending coronary artery appears occluded by thrombosis.

**Fig. 3:**
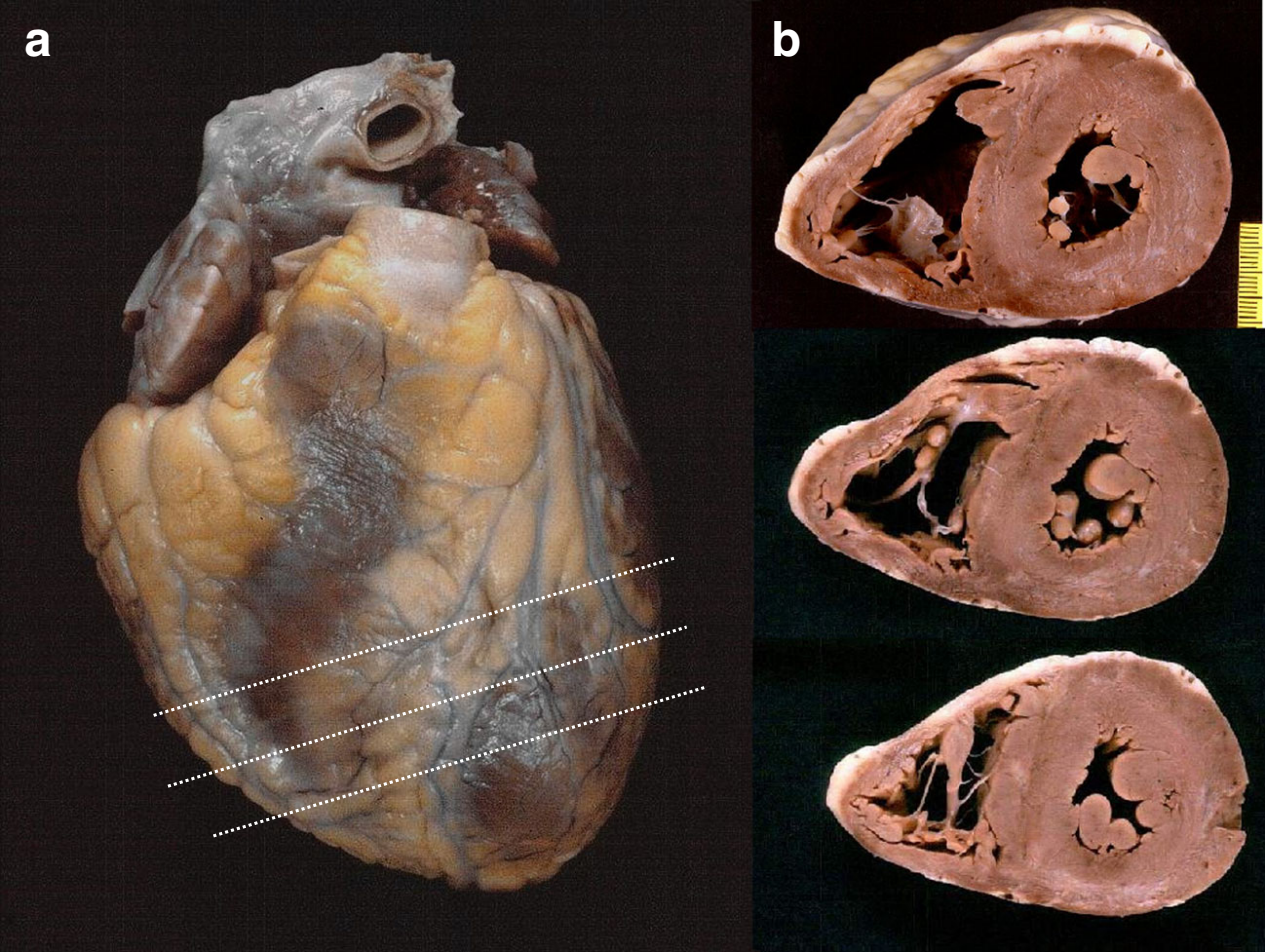
Short axis cross sectioning of the heart specimen from mid-ventricular to apical levels. **a** gross view of the specimen before cross sectioning. **b** transverse sections of the heart at the three different levels as represented in **a**

1. Check the pericardium, open it, and explore the pericardial cavity.
2. Check the anatomy of the great arteries and open the pulmonary artery in situ to identify embolus, before transecting them 3 cm above the aortic and pulmonary valves.
3. If congenital heart disease is suspected, the heart and lungs are best removed en bloc by cutting the pericardium from the diaphragm and transecting the inferior vena cava, aorta, and esophagus.
4. If aortic dissection is suspected, the continuity of the ascending aorta with aortic arch and descending thoraco-abdominal aorta up to the iliac bifurcation should be maintained.
5. Check and transect the pulmonary veins. Transect the superior vena cava 2 cm above the point where the crest of right atrial appendage meets the superior vena cava (to preserve sinus node). Transect the inferior vena cava close to the diaphragm.
6. Open the right atrium from the inferior vena cava to the apex of the appendage. Open the left atrium between the superior pulmonary veins and then to the atrial appendage. Inspect the atrial cavities and the inter-atrial septum and determine whether the foramen ovale is patent. Examine the mitral and tricuspid valves (or valve prostheses) from above and check the integrity of the papillary muscles and chordae tendineae.
7. Inspect the aorta, the pulmonary artery, and the aortic and pulmonary valves (or valve prosthesis) from above.
8. Check coronary arteries:
  - examine the size, shape, position, number, and patency of the coronary ostia (Fig. 1);
  - assess the size, course, and “dominance” of the major epicardial arteries;
  - make multiple transverse cuts at 3-mm intervals along the course of the main epicardial arteries, including branches such as the diagonal and obtuse marginal, and check patency (Fig. 2a, b);
  - heavily calcified coronary arteries should be removed intact, decalcified, and opened transversely; sometimes, they can be examined in situ with transverse sections made with sharp scissors;
  - coronary artery segments containing a metallic stent should be referred intact to laboratories with facilities for resin embedding. This permits sectioning of the arterial wall with the stent in situ, preserving their anatomic relationships [27]. In the early hours or days, after implantation, stents can be opened longitudinally, removed, and checked for thrombus. As an alternative, a scalpel can be used to cut down onto the stented vessel to inspect the stent lumen; thrombus filling the lumen can be removed for standard histology. Electrochemical and other novel techniques for dissolution of metallic and plastic stents that permit subsequent embedding of arteries in paraffin have been described [28]. These allow thin sectioning, special staining, and immunohistochemistry and are currently under investigation [29]. Radiology of the stented vessel before any further study is recommended;
  - coronary artery bypass grafts (saphenous veins, internal mammary arteries, radial arteries, etc) should be carefully examined with transverse cuts. The proximal and distal anastomoses should be examined with particular care. Side branch clips or sutures may facilitate their identification, particularly internal mammary grafts.
9. Make a complete *transverse* (short-axis) cut of the heart at the mid-ventricular level and then further parallel transverse slices of ventricles at 1 cm intervals towards the apex. Assess these slices carefully looking for changes in the cut surface of the myocardium and the endocardium of the ventricular cavities. (Fig. 3a, b). We strongly recommend not to dissect the myocardium with slices parallel to the endo-epicardial surfaces.
10. Triphenyltetrazolium (TTC) staining can be used in the autopsy room for preliminary diagnosis of acute myocardial infarction. A 1-cm thick ventricular slice should be selected and immersed in neutral TTC solution for 15–20 min at 37 °C.
11. Once emptied of blood, the following measurements should be always obtained:
  - Total heart weight: assess weight of heart against tables of normal weights by age, gender, and body weight*/*height [20–23].
  - Wall thickness: carefully inspect the endocardium, measure the thickness of mid-cavity free wall of the left ventricle and right ventricle and the interventricular septum (excluding trabeculae). Compare the measurements against tables of normal thickness by age, gender, and body weight [20–22]. Additional measurements can be made to estimate of the dimensions of chambers. Transverse size is best calculated as the distance from the obtuse to the acute margin in the posterior atrio-ventricular sulcus. The longitudinal size is obtained from a measurement of the distance between the crux cordis (i.e., the point at which the atrio-ventricular sulcus meets the posterior interventricular sulcus) and the apex of the heart on the posterior aspect.
12. Dissect the remainder of right and left ventricles in the basal half of the heart in the direction of flow of blood. Complete the examination of atrial and ventricular septa, atrio-ventricular valves, ventricular inflows and outflows, and semilunar valves. If there is ECG evidence of ventricular pre-excitation, the atrio-ventricular rings should be maintained intact.

#### Referral of hearts to specialized centers

Best practice is that the entire heart is retained and sent to a specialized center for an expert opinion [30]. The referring pathologist should complete steps 1–7, including making a transverse apical section of the heart and emptying the heart of blood. Tissues, blood, and other fluids for additional testing should be taken before fixing the heart in formalin (see below).

If the heart cannot be retained, it is essential that extensive photographic documentation is made, indicating where individual blocks are taken. At least one transverse section of the heart including the left and right ventricle should be retained for further examination.

#### Hearts from patients after surgery or percutaneous interventions

Pathologists should be familiar with recent developments in surgical and interventional treatment of cardiovascular diseases. These include new generations of coronary stents, ablation techniques, closure devices, and transcatheter valves, some of which create “novel” pathology with an inherent risk of SD. Discussion with the surgeon and/or interventional cardiologist before, or even during the autopsy, is again therefore of the utmost importance [31]. SD after surgery for complex congenital heart disease, including grown-up congenital heart disease, is an increasingly common event. The pathologist must understand the specific nature of the congenital anomaly involved and the exact operative procedures that were performed. In cases of congenital heart diseases, the heart-lung block should ideally be kept intact, fixed in formalin, and sent to a specialized cardiac pathology service [30].

Examination of the heart can be very difficult when extensive pericardial adhesions have developed after previous cardiac surgery. Nevertheless, specimens should be handled carefully in order to prevent damage or dislodgment of prostheses, stents, catheters, arterial conduits, and anastomoses or vascular grafts. A plain X-ray of the heart including surrounding fibrous adhesions can be very helpful in the localization of prosthetic devices. After coronary artery surgery, a post-mortem angiogram to visualize the patency of grafts and their run-off into distal native arteries could be helpful.

### The standard histologic examination of the heart

**Fig. 4:**
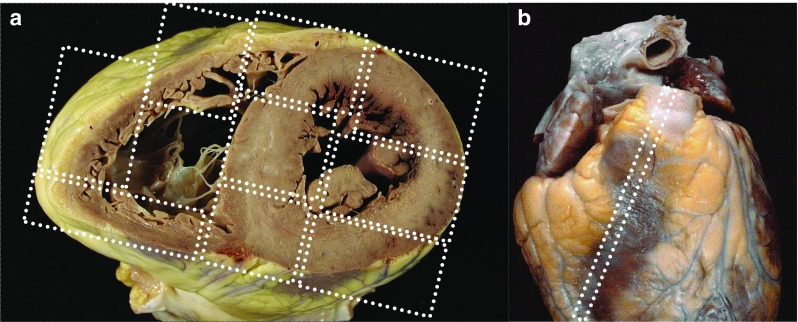
**a** Sampling of the myocardium with several transmural blocks, circumferentially along the left ventricle, septum and right ventricle. **b** An additional sampling of the right ventricular outflow tract myocardium can be also taken

- Myocardium: take mapped labeled blocks from a representative transverse slice of the ventricles to include the free wall of the left ventricle (anterior, lateral, and posterior), the ventricular septum (anterior and posterior), and the free wall of the right ventricle (anterior, lateral, and posterior), as well as right ventricular outflow tract (Fig. 4a, b). Left ventricular myocardial sections should include the papillary muscles. Additionally, one block from each atria and any area with significant macroscopic abnormalities should be taken. H & E and a connective tissue stain (Elastic van Gieson, trichrome or Sirius red) are standard. Other special stains and immunohistochemistry should be performed as required.
- Coronary arteries: in the setting of coronary artery disease, the most severe focal lesions should be sampled for histology in labeled blocks and stained as above.
- Other cardiac samples (such as valvular tissue, pericardium, and aorta) as indicated.
- If the clinical history or ECG tracing suggest an unusual conduction abnormality, the heart should be referred intact to a specialist center. The selection of blocks for the histological assessment of the conduction system requires particular expertise and serial sections are usually required.

#### Other tissues for histological examination

Specimens from the major organs should be taken routinely and stained with H & E and appropriate other stains.

### Photographic documentation

Modern methods of digital photography and video recording allow accurate documentation of normal and abnormal anatomy [32]. They are an essential part of a forensic autopsy but are also valuable in natural SD. In SCD, indefinite storage of entire hearts is not feasible and in many countries, organ retention is subject to either legal or ethical restriction. The written description of the heart, along with detailed photographs, forms the permanent record of the case. We believe that any gross finding relevant to the final diagnosis should be photographed and stored in the laboratory information system. This is of particular importance when the heart cannot be retained.

We recommend that photographs are taken before histological blocks are dissected from the heart, that they should contain an identification number and a ruled reference scale. Photographs should also be taken before any cardiac device is removed. Finally, the sites of the sections taken for histology should be photographed or described in detail in the report. This may help in the correct interpretation of the histological findings.

### Post-mortem imaging

In cardiovascular deaths, traditional plain X-ray images and post-mortem computed tomography (PMCT) are helpful in visualizing hemopericardium, calcified plaques, and valves, and in identifying and locating cardiovascular devices. Angiographic methods provide detailed views of the distribution pattern of epicardial coronary arteries, information on extent and location of arterial stenoses and obstructions, and specifically, the patency of stents and bypass grafts [33, 34]. Plain X-ray image and coronary angiography practiced on isolated hearts are easy and more accessible methods than whole body imaging and additional post-mortem coronary angiograms can be made immediately after removal of the heart using a small cabinet system [33]. Whole body imaging by PMCT and post-mortem magnetic resonance imaging (PMMR), with or without angiography, are increasingly used, especially in forensic practice. In addition, religious objections to invasive autopsies have led to a demand for minimally invasive radiological methods, with or without post-mortem biopsy of selected organs. There are advantages and disadvantages to different whole body imaging methods, which are related to the logistics, costs, and image quality. Some reports suggest that in natural deaths these methods are less accurate than a standard autopsy, especially in cardiovascular deaths [35–38]. However, PMCT angiography does allow the evaluation of vessels, including coronary arteries [34, 39, 40]. PMMR is currently available in only a few academic centers. Myocardial abnormalities can be visualized but the images may be modified by post-mortem changes in heart muscle [41, 42]. At present, the AECVP task force does not recommend the routine use of post-mortem imaging in the investigation of SD, but recognizes the added value in specific causes of death.

### Further post-mortem laboratory tests

Additional laboratory analyses as toxicology, chemistry, microbiology, and genetic testing may be required at the time of autopsy, or at a later stage. National and international recommendations for forensic autopsy sampling and subsequent analyses should be applied [see Table 1]. In the absence of suitable on-site facilities, stored material should be sent to specialized local or regional centers. National legal and ethical aspects must be considered, in particular for post-mortem genetic testing.

**Table 1:** Sampling for further laboratory tests in SD

Sample	Quantity	Technical aspects and storage	Analyses
Peripheral venous blood	10–20 ml	Potassium oxalate and sodium fluoride as preservatives, stored at 4° when analyzed promptly after autopsy, otherwise frozen at − 20°	Toxicology
Peripheral venous blood^a^	10 ml	EDTA, frozen optimally at − 80 °C	Genetic analyses, Virology
Serum	5–10 ml	From peripheral venous blood centrifuged directly after sampling, stored at 4° when analyzed promptly after autopsy, otherwise frozen at − 20°	Toxicology, Chemistry
Vitreous humor	5–10 ml	Stored at 4° when analyzed promptly after autopsy, otherwise frozen at − 20°	Toxicology, Chemistry
Hair	Strands (apx 0.2 g)	From the vertex posterior, cut closely to the scalp, pulled out and containing hair bulbs if possible, stored in an aluminum foil, an envelope or in a plastic tube at ambient temperature	Toxicology
Urine	10–20 ml	Stored at 4° when analyzed promptly after autopsy, otherwise frozen at − 20°	Toxicology, Chemistry
Bile	All	Stored at 4° when analyzed promptly after autopsy, otherwise frozen at − 20°	Toxicology
Pericardial/Cerebrospinal fluids	5–10 ml	Stored at 4° when analyzed promptly after autopsy, otherwise frozen at − 20°	Toxicology, Chemistry
Gastric contents	Whole	Stored at 4° when analyzed promptly after autopsy, otherwise frozen at − 20°	Toxicology
Other tissue samples^b^	25–50 g	Stored at 4° when analyzed promptly after autopsy, otherwise frozen at − 20°	Toxicology
Heart/spleen	5 g	Optimally frozen at − 80 °C, otherwise RNA later at room temperature; paraffin-embedded tissue are not optimal but might be considered	Genetic analyses, Virology
Blood or tissue culture	5–10 g	Recipients with specific media, depend on local laboratory	Microbiology, Genetic analyses
Myocardium and other tissue samples^c^	1 × 1 × 1 mm	Karnovsky fixative, stored at 4°	Ultrastructural analysis (electron microscopy)

Post-mortem specimens should be collected as soon as possible. The national and international quality management guidelines for standard autopsy sampling include several body fluids and tissues and describe appropriate technical methods. However, the extent of the sampling procedure will vary considerably from case to case, depending on autopsy results, circumstances of death, clinical data, requests, legal aspects, and availability. For most samples, disposable hard plastic or glass tubes are recommended

^a^or other location if peripheral blood not available

^b^liver, brain, lung, kidney, subcutaneous fat, and skeletal muscle

^c^in the suspicion of rare mitochondrial, storage, infiltrative diseases (tissues can be fixed in 2.5% glutaraldehyde only for short periods and must be processed promptly)

#### Toxicology

In all cases of SD, even if the heart is found to be abnormal at macroscopic and/or microscopic examination, the possibility that an illicit drug or a prescribed drug may have triggered the death, acting as additional factor to the anatomical substrate, should be carefully considered [43]. Many prescribed drugs or illicit substances have acute or long-term cardiovascular toxicity [44, 45]. Some prescribed drugs, such as antipsychotics, can modify the QT intervals or provoke myocarditis. Genetic factors play a role in the individual response to some medications and gene–drug interaction should be considered, especially with QT interval-acting drugs [46]. Illicit drugs, especially cocaine, have well described acute and chronic effects on the cardiovascular system [47–49]. However, many other recreational drugs can be involved in SD and novel “designer” drugs are continually appearing on the streets. As some of these cannot be detected by routine assays, close collaboration with toxicologists is necessary [50].

#### Chemistry

Post-mortem biochemistry is valuable in the diagnosis of deaths due to metabolic disorders, including alcoholic and diabetic ketoacidosis, electrolyte disorders, and anaphylaxis, when there is a prolonged stress response (hypothermia, starvation), and might be useful in the diagnosis of disease processes such as inflammation, early myocardial infarction, and sepsis [51–53].

#### Microbiology

Myocarditis is a well-recognized cause of SCD, especially in the young. The cause can be infective (mostly viral), toxic, allergic, drug induced, or immune. In lymphocytic myocarditis, molecular techniques such as polymerase chain reaction (PCR) on both myocardial tissue and blood are the gold standard for the diagnosis of viral myocarditis [54, 55]. The viral genome load should be quantified, particularly for viruses such as PVB19, which is frequently present as an innocent bystander [56]. Systemic sepsis and organ infection, including endocarditis, are uncommon, but well-recognized causes of SD. Cultures of blood, cerebrospinal fluid, spleen, or cardiac vegetation should be considered [57].

#### Genetic testing

Many cardiovascular diseases causing SCD have a genetic background. Some are associated with well-defined structural abnormalities but in others, the heart may be macroscopically and microscopically normal. The likelihood that a case of unexplained SCD is caused by an underlying inherited disorder has led to the emerging role of genetic testing of DNA obtained at autopsy (also called “molecular autopsy,” but the term post-mortem genetic testing is preferable) [55, 58]. Thus, pathologists play an important role in the identification of families at risk, by reporting whether it is recommended to refer first-degree family members for clinical screening and/or to perform additional post-mortem genetic testing with cascade genetic screening, based upon the autopsy findings [59–62]. In Table 2, diseases are listed in whom cardiological assessment of relatives is recommended according to international guidelines [58, 59, 63–75]. Only a minority of these studies specifically gives recommendations for genetic testing in the DNA of the deceased probands. In particular, according to the ESC guidelines [7], targeted post-mortem genetic analysis of potentially disease-causing genes should be considered in all SD victims in whom a specific inheritable channelopathy or cardiomyopathy is suspected (Class of recommendation IIa, level of evidence C). However, in most of European countries, post-mortem genetic testing is performed only if there is genetic counseling of family members. After genetic counseling, it can be decided to perform genetic testing of the deceased in the context of the family screening.

**Table 2:** Cardiogenetic studies of family members: class of recommendations based on autopsy findings of the proband

Finding autopsy	Age limit	Possible mutated genes	Class of recommendation for referral of first-degree family members for clinical/genetic counseling	Level of evidence [References]
Unknown/Uncertain cause of SCD	≤ 40	Mainly ion channels genes	I	C [58] [59, 63, 64]
Hypertrophic cardiomyopathy	No limit	Sarcomeric and other disease related genes	I	C [59, 65]
Arrhythmogenic cardiomyopathy	No limit	Desmosomal and other disease-related genes	I	C [66]
Dilated cardiomyopathy	No limit	Sarcomeric, cytoskeleton and other disease-related genes	I	C [59]
Premature atherosclerosis	Men < 40 Women< 50	Familial hypercholesterolemia genes	IIa	C [67, 68]
Thoracic aortic aneurysm / dissection / rupture with medial degeneration	Unknown	Syndromic and non-syndromic aortic aneurysm-related genes	I	C [69–71]
Spontaneous coronary artery dissection	No limit	Connective tissue disease-related genes	IIa	C [72]
Pulmonary embolism	Unknown	Hereditary thrombophilia genes	IIb^a^	C [73]
SUDEP	Unknown	Overlap with ion channel-related genes	IIa	C [74, 75]

Age limit refers to age of autopsy patient. *SUDEP* sudden unexpected death in epilepsy

Class of recommendation: *I* is recommended, *IIa* can be useful, *IIb* may be considered, *III* is not recommended

Level of evidence: *A* data derived from multiple randomized clinical trials or meta-analyses, *B* data derived from a single randomized clinical trial or large non-randomized studies. *C* consensus of opinion of the experts and/or small studies, retrospective studies, registries

^a^may be considered especially for patients without known risk factors for pulmonary embolism

### Formulation of a diagnosis and the clinico-pathological summary

The autopsy report should conclude with a clear clinico-pathological summary of the major positive findings and their relationship to the cause of death (epicrisis). As far as possible, this should relate the pathological findings to the clinical history, the circumstances of the death, to any investigation performed close to the time of the death and to the results of all laboratory analyses. In the majority of SCDs, a clear pathological cause can be identified, albeit with varying degrees of confidence. When the morphological findings, and the results of additional analyses, do not indicate a single specific diagnosis, the full range of possible differential diagnoses should be discussed. These should be ranked in order of probability [76, 77] and whenever possible, the most likely underlying cause should be stated [78].

It is important to accept that *different degrees of certainty* exist in defining the cause-effect relationship between the cardiovascular findings and the SD event. Table 3 lists the commonest substrates of SCD, classifying each as *certain*, *highly probable,* or *uncertain*. In the probable, and especially the uncertain categories, each case should be considered on its individual merits. The clinical history, the circumstances of death, and the results of ancillary analyses may influence the decision-making process.

**Table 3:** Certainty of diagnosis of cardiovascular substrates of SCD at post-mortem

	Certain	Highly probable	Uncertain
Coronary artery disease (native coronary arteries/stent/grafts/cardiac allograft)	Myocardial infarction, acute (any cause)	Chronic ischemic heart disease (ischemic scar, any cause)	Anomalous RCA origin from the left sinus with inter-arterial course
	Acute coronary occlusion (atherothrombosis, arteritis, dissection or embolism, cardiac allograft vasculopathy)	Atherosclerotic plaque with coronary luminal stenosis > 75%	Wrong aortic sinus coronary artery anomalies without inter-arterial course
	Coronary ostia mechanical obstruction (aortic or valve prosthesis, tumor, vegetation)	Anomalous LCA origin from the right sinus with inter-arterial course	High take-off from the tubular portion
	Anomalous origin of the coronary artery from the pulmonary trunk		Anomalous LCx origin from the right sinus or coronary artery
			Anomalous LAD origin with course anterior to the pulmonary artery
			Coronary ostia plication
			Intra-myocardial course of LAD (myocardial bridge)
			Small vessel disease
Myocardial diseases	Acute diffuse myocarditis (any morphological type)	Hypertrophic CM	Focal myocarditis
		Arrhythmogenic CM	Idiopathic LV hypertrophy
		Dilated CM	
		Idiopathic fibrosis (non-ischemic LV scar)	Hypertensive heart disease
		Multifocal myocarditis	Hypertrabeculation (non-compacted) myocardium
		Sarcoidosis	
		Storage diseases	
		Amyloidosis	
Native /prosthetic valves diseases	Mitral valve papillary muscle or chordae tendineae rupture with mitral valve incompetence and pulmonary edema	Calcific aortic valve stenosis with LV hypertrophy and fibrosis	Moderate aortic valve sclerosis without LV hypertrophy/ mitral annular calcification
	Thrombotic block or endocarditis vegetations on valve prosthesis	Myxoid degeneration of the mitral valve (prolapse) with atrial dilatation or LV myocardial fibrosis and intact chordae	Dystrophic calcification of the membranous septum (+/− mitral annulus/aortic valve)
	Laceration/Dehiscence/Leaflet escape of valve prosthesis with acute valve incompetence		Aortic insufficiency (dilated aortic annulus)
			Myxoid degeneration of the mitral valve (prolapse) without atrial dilatation or LV fibrosis and intact chordae
Conduction system diseases		AV node cystic tumor	Hemorrhage of the sub-aortic septum
		Purkinje cell hamartoma	Fibrosis of RBB and LBB (Lenègre disease)
		Sarcoidosis of the AV conduction system	
		Surgical stiches, perimembranous	
Congenital heart diseases	BAV and/or Isthmic coarctation with aortic dissection	Tetralogy of Fallot, surgical repair +/−pulmonary valve incompetence and RV dilatation	CHD with septal defect, no obstructive pulmonary vascular disease, repaired or unrepaired
		Congenital aortic stenosis (supra-, sub- or valvular) or isthmic coarctation with LV hypertrophy and fibrosis	Any other unrepaired CHD
		CHD with septal defects, repaired or unrepaired, and obstructive pulmonary vascular disease (Eisenmenger syndrome)	
		CHD with perimembranous VSD, postero-inferior rim stich	
		Corrected TGA (unrepaired)	
		Ebstein anomaly	
		Atrio-ventricular anomalous pathway (Kent fascicle)	
		TGA, atrial or arterial switch operation	
		CHD with RV-pulmonary artery conduit repair	
		CHD with univentricular or one and half repair	
		Ross operation	
Others	Massive pulmonary embolism Hemopericardium (aorticrupture/dissection or cardiac rupture)	Intramural ventricular/septal tumor	Atrial septum lipoma
	Myxoma or other tumor/thrombus obstructing a valve orifice		Congenital partial absence of pericardium

AV atrio-ventricular, BAV bicuspid aortic valve, CHD congenital heart disease, CM cardiomyopathy LAD left anterior descending, LBB left bundle branch, LCA left coronary artery, LCx left circumflex, LV left ventricle, RBB right bundle branch, RCA right coronary artery, RV right ventricle, TGA transposition of the great arteries, VSD ventricular septal defect

Finally, there are myocardial diseases in which the border between physiological and pathological changes is poorly defined. Some of these *diagnostic gray zones* are described in Table 4 [64, 79–81]. In everyday practice, pathologists should make a detailed macroscopic and microscopic description of their findings, without implying a cause and effect relationship. If there is real doubt as to whether the changes are physiological or pathological, an expert opinion should be sought.

**Table 4:** The gray zone between normal and/or secondary changes and pathologic changes of the myocardium

Changes in the range of normality or secondary changes	Pathologic changes	Comments
Fatty infiltration of the right ventricular wall	Arrhythmogenic cardiomyopathy	Massive fatty infiltration of the right ventricle, without any evidence of replacement-type fibrosis and myocyte degeneration, should not be considered a diagnostic finding of arrhythmogenic cardiomyopathy, especially in obese, elderly people and people with alcohol abuse
Exercise-induced left ventricular hypertrophy (athlete’s heart)	Hypertrophic cardiomyopathy	An enlarged left ventricular cavity with increased wall thicknesses up to 13–14 mm is present in more than one third of highly trained athletes. Detailed histology essential
Focal myocardial disarray without hypertrophy	Hypertrophic cardiomyopathy without hypertrophy	Macroscopic changes are not always present in hypertrophic cardiomyopathy. Isolated myocardial disarray confined to the antero-septal and postero-septal junctions should be considered physiologic. For a confident diagnosis, additional findings, such as interstitial and/or replacement fibrosis and abnormal intramyocardial blood vessels should be searched for
Scattered inflammatory foci with or without small foci of fibrosis	Focal myocarditis	In the absence of myocyte necrosis, small foci of inflammatory cells (even after immunohistochemistry), are not sufficient evidence of myocarditis. Scattered small foci of fibrosis are also insignificant.
Circumferential, subendocardial myocardial ischemia+/− hemorrhage after resuscitative maneuvers	Regional or circumferential, sub-endocardial myocardial ischemia without resuscitative maneuvers	Ischemic changes of the myocardium, particularly when sub-endocardial and diffuse require exclusion of prolonged resuscitative maneuvers

The clinical history, the autopsy findings, and the results of all ancillary examinations must be considered together when estimating the certainty of a cause of death. Interpretation of results of toxicology can be difficult. Quantification of drug levels, rather than their simple identification, is essential. Specific expertise may be necessary to state whether the amount of a particular drug is within the therapeutic range or potentially or definitely toxic. The information obtained from toxicological analyses depends not only on the presence of the substance in the body but also on analytical methods used. These include screening procedures, confirmation procedures, and specific methods. Many analytical pitfalls, especially for screening procedures by immunoassay, should be considered [82]. Moreover, substantial changes can occur in blood drug levels between the time of death and the autopsy. These changes are not easily identifiable by post-mortem sampling and toxicological analyses. They are related to drug degradation, neo-formation, artefactual formation, post-mortem redistribution, and individual pharmacogenomics [46]. The interpretation of post-mortem chemistry is limited by the lack of knowledge on defined reference ranges in life, by the effects of autolysis and other post-mortem changes and time interval between death and sampling [51, 53].

Finally, there are deaths that remain unexplained after careful macroscopic, microscopic, and laboratory analyses (referred as SADS by the clinicians) [83–85]. We strongly suspect that the numbers of these unexplained deaths have been underestimated in the past and that there are many borderline cases. There is increasing evidence that SCD in these instances might be due to inherited ion channel disorders, which produce well-defined abnormalities in a basal or effort ECG. In this setting, the availability of ECG tracings may be crucial for the diagnosis and molecular studies are essential. First-degree relatives should be referred to a department with specific expertise in inherited cardiac conditions for clinical screening and, when indicated, subsequent genetic analysis. High-throughput approaches, such as massive parallel or next-generation sequencing, enable rapid analysis of many genes at low cost and with a higher success rate than with Sanger technology. In a SCD victim, particularly in the absence of a clear phenotype, the mere identification of a genetic defect is not sufficient to establish a diagnosis and state the cause of death [86]. A multidisciplinary approach is essential. Genetic information should always be interpreted in the clinical context of the deceased and his/her family and the autopsy findings [61, 87].

## Conclusions

Autopsy represents the first and last opportunity to make an accurate diagnosis in SCD. Our recommended method of investigation has been updated and includes not only a protocol for examination of the heart and histological sampling, but, when necessary, for toxicology, microbiology, biochemistry, and molecular investigation. Our recommendations apply not only to university medical centers and regional hospitals but also to all people practicing pathology and forensic medicine. If these guidelines are adopted throughout the European Union, they will lead to improvements in standards of practice and will allow meaningful comparisons between different communities and regions. Most importantly, they will facilitate the identification of novel causes, and emerging patterns of diseases, causing SCD.

The writing committee strongly supports the establishment of regional multidisciplinary expert networks. These should include pathologists, cardiologists, and geneticists, working in collaboration with microbiologists, toxicologists, and radiologists. The major purposes of these networks are to improve the diagnosis of SCD and to identify and organize preventive strategies for family members in the setting of genetic abnormalities.

## References

[1] Basso C (2008). Guidelines for autopsy investigation of sudden cardiac death. Virchows Arch.

[2] Shojania KG (2003). Changes in rates of autopsy-detected diagnostic errors over time: a systematic review. JAMA.

[3] Turnbull A, Osborn M, Nicholas N (2015). Hospital autopsy: endangered or extinct?. J Clin Pathol.

[4] van den Tweel JG, Wittekind C (2016). The medical autopsy as quality assurance tool in clinical medicine: dreams and realities. Virchows Arch.

[5] Goldstein S (1982). The necessity of a uniform definition of sudden coronary death: witnessed death within 1 hour of the onset of acute symptoms. Am Heart J.

[6] Virmani R, Burke AP, Farb A (2001). Sudden cardiac death. Cardiovasc Pathol.

[7] Priori SG (2015). ESC guidelines for the Management of Patients with ventricular arrhythmias and the prevention of sudden cardiac death: the task force for the management of patients with ventricular arrhythmias and the prevention of sudden cardiac death of the European Society of Cardiology (ESC). Endorsed by: Association for European Paediatric and Congenital Cardiology (AEPC). Eur Heart J.

[8] Mendis S, Puska P, Norrving B (2011) Global Atlas on cardiovascular disease prevention and control, WHO, Editors: WHO; World Heart Federation; World Stroke Organization

[9] Kong MH (2011). Systematic review of the incidence of sudden cardiac death in the United States. J Am Coll Cardiol.

[10] Myerburg RJ, Junttila MJ (2012). Sudden cardiac death caused by coronary heart disease. Circulation.

[11] Myerburg RJ, Kessler KM, Castellanos A (1992). Sudden cardiac death. Structure, function, and time-dependence of risk. Circulation.

[12] Thiene G, Rizzo S, Basso C (2016) In: Butany J (eds) Cardiovascular pathology, 4th Edition. Elsevier, Oxford p, 361–433

[13] Myerburg R, Wellens H, Priori S, Zipes D (2006). Epidemiology of cardiac arrest. Sudden cardiac death.

[14] Zipes DP (2006). ACC/AHA/ESC 2006 Guidelines for Management of Patients With Ventricular Arrhythmias and the Prevention of Sudden Cardiac Death: a report of the American College of Cardiology/American Heart Association Task Force and the European Society of Cardiology Committee for Practice Guidelines (writing committee to develop Guidelines for Management of Patients With Ventricular Arrhythmias and the Prevention of Sudden Cardiac Death): developed in collaboration with the European Heart Rhythm Association and the Heart Rhythm Society. Circulation.

[15] Chugh SS (2008). Epidemiology of sudden cardiac death: clinical and research implications. Prog Cardiovasc Dis.

[16] Deo R, Albert CM (2012). Epidemiology and genetics of sudden cardiac death. Circulation.

[17] Priori SG (2001). Task force on sudden cardiac death of the European Society of Cardiology. Eur Heart J.

[18] Myerburg R, Castellanos A, Saunders W (2001). Cardiac arrest and sudden cardiac death. Heart disease: a textbook of Cardiovascular Medicine.

[19] Brinkmann B (1999). Harmonisation of medico-legal autopsy rules. Int J Legal Med.

[20] Kitzman DW (1988). Age-related changes in normal human hearts during the first 10 decades of life. Part II (maturity): a quantitative anatomic study of 765 specimens from subjects 20 to 99 years old. Mayo Clin Proc.

[21] Scholz DG (1988). Age-related changes in normal human hearts during the first 10 decades of life. Part I (Growth): A quantitative anatomic study of 200 specimens from subjects from birth to 19 years old.[erratum appears in Mayo Clin Proc 1988 Jun;63(6):637]. Mayo Clinic Proc.

[22] Schulz DM, Giordano DA (1962). Hearts of infants and children. Weights and measurements. Arch Pathol.

[23] Vanhaebost J (2014). New reference tables and user-friendly internet application for predicted heart weights. Int J Legal Med.

[24] Ashwell M, Gibson S (2016). Waist-to-height ratio as an indicator of ‘early health risk’: simpler and more predictive than using a ‘matrix’ based on BMI and waist circumference. BMJ Open.

[25] Kirkpatrick JN (2007). Postmortem interrogation and retrieval of implantable pacemakers and defibrillators: a survey of morticians and patients. J Cardiovasc Electrophysiol.

[26] https://www.gov.uk/drug-device-alerts/medical-device-alert-implantable-cardioverter-defibrillators-icds-disable-all-high-voltage-shock-therapies-before-you-remove-icd. Alerts and recalls for drugs and medical devices 2008 01.03.2017]

[27] Rippstein P (2006). Comparison of processing and sectioning methodologies for arteries containing metallic stents. J Histochem Cytochem.

[28] Bradshaw SH (2009). A practical method to rapidly dissolve metallic stents. Cardiovasc Pathol.

[29] Fishbein I (2016). Paraffin processing of stented arteries using a postfixation dissolution of metallic and polymeric stents. Cardiovasc Pathol.

[30] Thiene G (2010). AECVP and SCVP 2009 recommendations for training in cardiovascular pathology. Cardiovasc Pathol.

[31] Houser SL (2009). The operated heart at autopsy.

[32] Rampy BA, Glassy EF (2016). Pathology gross photography: the beginning of digital pathology. Clin Lab Med.

[33] Kornegoor R (2009). Digitalization of post-mortem coronary angiography. Histopathology.

[34] Grabherr S, Grimm JM, Heinemann A (2016). Atlas of postmortem angiography.

[35] Weustink AC (2009). Minimally invasive autopsy: an alternative to conventional autopsy?. Radiology.

[36] Roberts ISD (2012). Post-mortem imaging as an alternative to autopsy in the diagnosis of adult deaths: a validation study. Lancet.

[37] Blokker BM (2016). Non-invasive or minimally invasive autopsy compared to conventional autopsy of suspected natural deaths in adults: a systematic review. Eur Radiol.

[38] Michaud K et al (2013) Postmortem imaging of sudden cardiac death. Int J Legal Med:1–11 10.1007/s00414-013-0819-6 23322013

[39] Roberts ISD, Traill ZC (2014). Minimally invasive autopsy employing post-mortem CT and targeted coronary angiography: evaluation of its application to a routine coronial service. Histopathology.

[40] Michaud K (2012). Evaluation of postmortem MDCT and MDCT-angiography for the investigation of sudden cardiac death related to atherosclerotic coronary artery disease. Int J Cardiovasc Imaging.

[41] Ruder TD, Thali MJ, Hatch GM (2014). Essentials of forensic post-mortem MR imaging in adults. Br J Radiol.

[42] Jackowski C (2013). Post-mortem cardiac 3-T magnetic resonance imaging: visualization of sudden cardiac death?. J Am Coll Cardiol.

[43] Bjune T et al (2017) Post-mortem toxicology in young sudden cardiac death victims: a nationwide cohort study*.* Europace 10.1093/europace/euw435 28339816

[44] Fischbach P (2017). The role of illicit drug use in sudden death in the young. Cardiol Young.

[45] Woosley RL, Romero K (2013). Assessing cardiovascular drug safety for clinical decision-making. Nat Rev Cardiol.

[46] van Noord C, Eijgelsheim M, Stricker BH (2010). Drug- and non-drug-associated QT interval prolongation. Br J Clin Pharmacol.

[47] Karch SB (2005). Cocaine cardiovascular toxicity. South Med J.

[48] Lucena J (2010). Cocaine-related sudden death: a prospective investigation in south-west Spain. Eur Heart J.

[49] Montisci M (2012). Anabolic androgenic steroids abuse and cardiac death in athletes: morphological and toxicological findings in four fatal cases. Forensic Sci Int.

[50] Tominaga M (2015). Efficacy of drug screening in forensic autopsy: retrospective investigation of routine toxicological findings. Leg Med (Tokyo).

[51] Belsey SL, Flanagan RJ (2016). Postmortem biochemistry: current applications. J Forensic Legal Med.

[52] Sabatasso S (2011). Sensitivity and specificity of NT-proBNP to detect heart failure at post mortem examination. Int J Legal Med.

[53] Palmiere C, Mangin P (2012). Postmortem chemistry update part I. Int J Legal Med.

[54] Basso C (2013). Classification and histological, immunohistochemical, and molecular diagnosis of inflammatory myocardial disease. Heart Fail Rev.

[55] Basso C (2010). Sudden cardiac death with normal heart: molecular autopsy. Cardiovasc Pathol.

[56] Nielsen TS (2014). The presence of enterovirus, adenovirus, and parvovirus B19 in myocardial tissue samples from autopsies: an evaluation of their frequencies in deceased individuals with myocarditis and in non-inflamed control hearts. Forensic Sci Med Pathol.

[57] Fernández-Rodríguez A (2015). How to optimise the yield of forensic and clinical post-mortem microbiology with an adequate sampling: a proposal for standardisation. Eur J Clin Microbiol Infect Dis.

[58] Semsarian C, Ingles J, Wilde AA (2015). Sudden cardiac death in the young: the molecular autopsy and a practical approach to surviving relatives. Eur Heart J.

[59] Ackerman MJ et al (2011) HRS/EHRA expert consensus statement on the state of genetic testing for the channelopathies and cardiomyopathies: this document was developed as a partnership between the Heart Rhythm Society (HRS) and the European Heart Rhythm Association (EHRA). Heart Rhythm 8(8):1308–1339 10.1016/j.hrthm.2011.05.020 21787999

[60] Nunn LM, Lambiase PD (2011). Genetics and cardiovascular disease—causes and prevention of unexpected sudden adult death: the role of the SADS clinic. Heart.

[61] Wilhelm M (2015). Sudden cardiac death in forensic medicine - Swiss recommendations for a multidisciplinary approach. Swiss Med Wkly.

[62] Wong LCH (2014). Cardiac evaluation of pediatric relatives in sudden arrhythmic death syndrome: a 2-center experience. Circ Arrhythm Electrophysiol.

[63] Bagnall RD (2016). A prospective study of sudden cardiac death among children and young adults. N Engl J Med.

[64] Papadakis M (2013). Sudden cardiac death with autopsy findings of uncertain significance: potential for erroneous interpretation. Circ Arrhythm Electrophysiol.

[65] Elliott PM (2014). 2014 ESC guidelines on diagnosis and management of hypertrophic cardiomyopathy: the task force for the diagnosis and Management of Hypertrophic Cardiomyopathy of the European Society of Cardiology (ESC). Eur Heart J.

[66] Marcus FI (2010). Diagnosis of Arrhythmogenic right ventricular cardiomyopathy/dysplasia. Proposed Modification of the Task Force Criteria. Circulation.

[67] Larsen MK et al (2012) Sudden cardiac death in young adults: environmental risk factors and genetic aspects of premature atherosclerosis. J Forensic Sci 57(3):658–662 10.1111/j.1556-4029.2011.02028.x 22220933

[68] Nordestgaard BG (2013). Familial hypercholesterolaemia is underdiagnosed and undertreated in the general population: guidance for clinicians to prevent coronary heart disease: consensus statement of the European Atherosclerosis Society. Eur Heart J.

[69] Erbel R (2014). 2014 ESC guidelines on the diagnosis and treatment of aortic diseases: document covering acute and chronic aortic diseases of the thoracic and abdominal aorta of the adult:the task force for the diagnosis and treatment of aortic diseases of the European Society of Cardiology (ESC). Eur Heart J.

[70] Halushka MK et al (2016) Consensus statement on surgical pathology of the aorta from the Society for Cardiovascular Pathology and the Association for European Cardiovascular Pathology: II. Noninflammatory degenerative diseases - nomenclature and diagnostic criteria. Cardiovasc Pathol 25(3):247–257 10.1016/j.carpath.2016.03.002 27031798

[71] Hiratzka LF (2010). 2010 ACCF/AHA/AATS/ACR/ASA/SCA/SCAI/SIR/STS/SVM guidelines for the diagnosis and management of patients with thoracic aortic disease. J Am Coll Cardiol.

[72] Henkin S (2016). Spontaneous coronary artery dissection and its association with heritable connective tissue disorders. Heart.

[73] De Stefano V, Rossi E (2013). Testing for inherited thrombophilia and consequences for antithrombotic prophylaxis in patients with venous thromboembolism and their relatives. A review of the guidelines from scientific societies and working groups. Thromb Haemost.

[74] Coll M (2016). Genetic investigation of sudden unexpected death in epilepsy cohort by panel target resequencing. Int J Legal Med.

[75] Goldman AM (2016). Sudden unexpected death in epilepsy genetics: molecular diagnostics and prevention. Epilepsia.

[76] Saukko P, Knight B (2016). Knight's forensic pathology fourth edition.

[77] Pollanen MS (2016). On the strength of evidence in forensic pathology. Forensic Sci Med Pathol.

[78] Thiene G, Corrado D, Basso C (2016). Sudden cardiac death in the young and athletes.

[79] Basso C, Thiene G (2005). Adipositas cordis, fatty infiltration of the right ventricle, and arrhythmogenic right ventricular cardiomyopathy. Just a matter of fat?. Cardiovasc Pathol.

[80] Hughes SE (2004). The pathology of hypertrophic cardiomyopathy. Histopathology.

[81] Tansey DK, Aly Z, Sheppard MN (2005). Fat in the right ventricle of the normal heart. Histopathology.

[82] Skopp G (2010). Postmortem toxicology. Forensic Sci Med Pathol.

[83] Behr E (2003). Cardiological assessment of first-degree relatives in sudden arrhythmic death syndrome. Lancet.

[84] Corrado D, Basso C, Thiene G (2001). Sudden cardiac death in young people with apparently normal heart. Cardiovasc Res.

[85] Fabre A, Sheppard MN (2006). Sudden adult death syndrome and other non-ischaemic causes of sudden cardiac death. Heart.

[86] Allegue C (2015). Genetic analysis of Arrhythmogenic diseases in the era of NGS: the complexity of clinical decision-making in Brugada syndrome. PLoS One.

[87] Miles CJ, Behr ER (2016). The role of genetic testing in unexplained sudden death. Transl Res.

